# Synthesis and Fluorescence Properties of 4-Cyano and 4-Formyl Melatonin as Putative Melatoninergic Ligands

**DOI:** 10.1021/acsomega.3c02518

**Published:** 2023-06-08

**Authors:** Silvia Bartolucci, Michele Retini, Fabiola Fanini, Daniele Paderni, Giovanni Piersanti

**Affiliations:** † Department of Biomolecular Sciences, University of Urbino Carlo Bo, Piazza del Rinascimento 6, 61029 Urbino, Pesaro and Urbino, Italy; ‡ Department of Pure and Applied Sciences, University of Urbino Carlo Bo, Via della Stazione 4, 61029 Urbino, Pesaro and Urbino, Italy

## Abstract

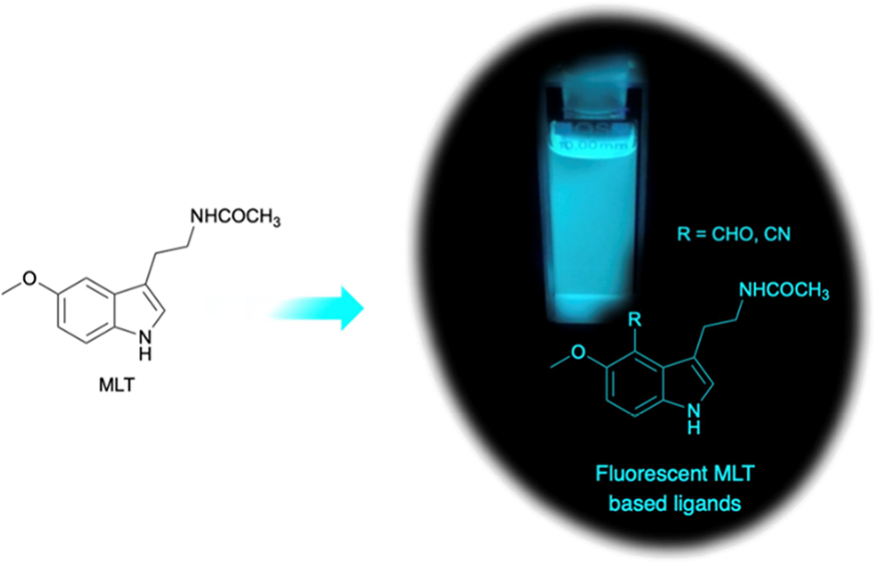

Fluorescent ligands are imperative to many facets of chemical biology and medicinal chemistry. Herein, we report the syntheses of two fluorescent melatonin-based derivatives as potential ligands of melatonin receptors. The two compounds, namely, 4-cyano and 4-formyl melatonin (4CN-MLT and 4CHO-MLT, respectively), which differ from melatonin by only two/three atoms that are very compact in size, were prepared using the selective C3-alkylation of indoles with *N*-acetyl ethanolamines involving the “borrowing hydrogen” strategy. These compounds exhibit absorption/emission spectra that are red-shifted from those of melatonin. Binding studies on two melatonin receptor subtypes showed that these derivatives have a modest affinity and selectivity ratio.

## 1 Introduction

Since most biomolecules are either intrinsically nonfluorescent or lack suitable fluorescence properties, one or multiple external fluorophores are frequently required in biological studies using fluorescence-based technology.^1−3^ Thus, fluorescent probes represent highly sensitive and safe tools for real-time exploration of the activity of biomolecules, visualizing cellular processes, studying ligand/receptor interactions, and more generally increasing the understanding of the pharmacology and physiological processes at the molecular level.^4−11^ However, the design and development of such probes are challenging, mainly due to the use of large-sized and interfering fluorophores/organic dyes attached to the ligand (often through a linker), low affinity/specificity of the probe for its target, and extensive nonspecific binding. One practical strategy to alleviate such issues has focused on the use of chromophores that are derived from naturally occurring ligands with a minimally sized reactive group and improved intrinsic optical properties. Considering the rapid progress of fluorescence technologies, effective small-molecule probes containing chromophores have been and still continue to be actively pursued as valuable tools.^12−14^

Melatonin (MLT) is an essential human hormone produced mainly by the pineal gland from the amino acid tryptophan; it plays a role in circadian rhythm regulation, acts as an antioxidant, and protects DNA. Despite the importance of MLT in the human body, studies on its interaction with biomolecules, considered explicitly, are quite limited in the literature. In particular, the action of MLT is mediated by two receptors, MT_1_ and MT_2_, which are members of the huge family of G protein-coupled receptors. These receptors are also implicated in biological processes other than circadian rhythm- and sleep-related disorders,^15,16^ although the potential benefits of MLT assumption in humans remain controversial.^17^ Despite the discovery of the high-affinity ligands,^18^ research on the pharmacology and the functionality/physiological impact of MLT receptors suffers from the lack of efficient fluorescent probes for these receptors that would allow, among others, studying the molecular dynamics of receptor activation at the molecular level. In addition, given the demanding technical set-up, hazards, and expense of using the 2-[^125^I]-MLT radioligand, fluorescence-based methodologies appear to be more desirable and easier to implement. However, developing such assays for MT_1_ and MT_2_ depends on the availability of fluorescent tracers, which has been challenging owing to their narrow ligand entry channel and small ligand-binding pocket. The fluorescently labeled melatonin receptor ligands described so far include melatonin-bodipy-fused analogues,^19^ 4-azamelatonin ligands with different fluorophores,^20^ melatonin coupled to the Cy3 cyanin fluorophore,^21^ and coumarin-based compounds,^22^ but data on their full pharmacological and functional properties are only fragmentary. To offer novel probes, we sought to improve the intrinsic optical properties of the 5-methoxyindole side chain of the endogenous ligand MLT, which possesses fluorescence properties that are unfortunately inappropriate for biological analysis (the main absorption band overlaps with those of the l-tyrosine and l-tryptophan residues already present in proteins), as the source of fluorescence after minor chemical modification, with extended conjugation of the structure that would exhibit absorption at longer wavelengths.^23−27^

Recent photophysical studies on a library of substituted indoles and tryptophans^28,29^ have demonstrated that the C4 position of the indole ring is special and that the substitution-induced spectral shift of a substituent may correlate with its electronic withdrawing strength. These reports prompted us to investigate extending the π-conjugation of the indole scaffold at the C4 position of MLT with a small-sized and strong electron-withdrawing group to obtain biologically compatible photophysical properties. Here, we report the design, synthesis, and spectroscopic studies of novel putative fluorescent ligands for MLT receptors that introduce a formyl moiety (CHO) or a cyano (CN) group on the C4 position of the MLT core, which affect the absorption and emission properties compared to the endogenous ligand MLT, without a detrimental effect on binding.^28,30^

## 2 Results and Discussion

**Scheme 1:**
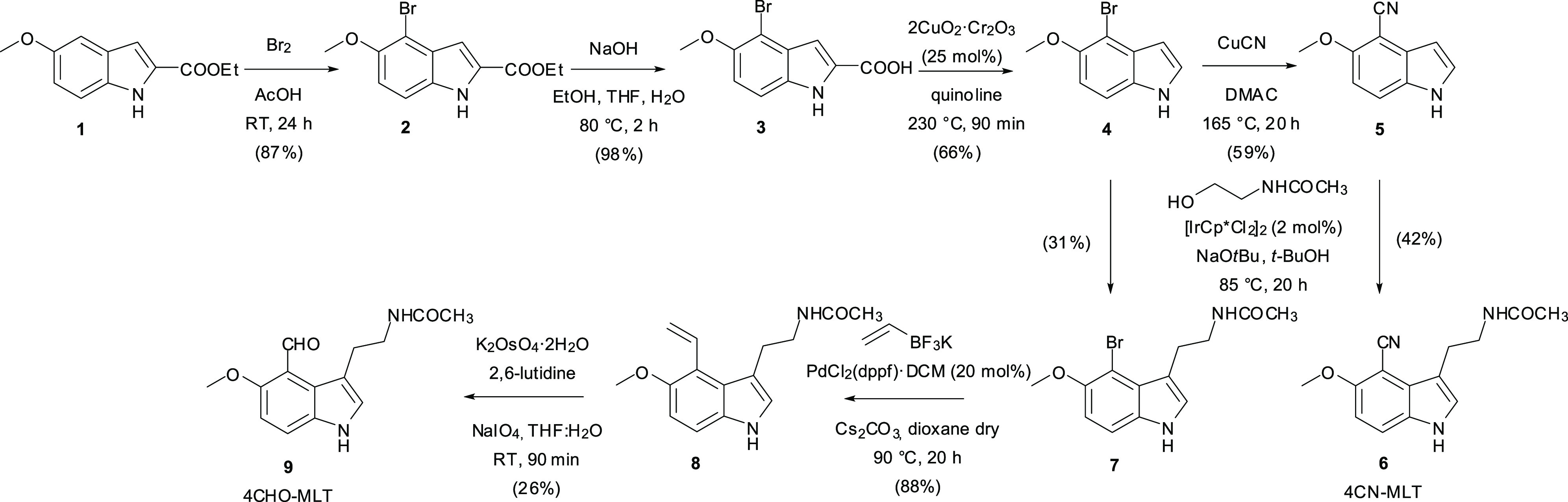
Synthesis of 4CN-MLT and 4CHO-MLT

Regarding the synthesis of the abovementioned new fluorescent ligands, we envisioned bromide **4** (Scheme 1) as a valuable key intermediate to install both the requisite side chain and the selected substituents in the C4 position of the indole ring. This intermediate was easily obtained from commercially available 5-methoxy-2-carboxyethyl indole (**1**) by C4-selective bromination (87% yield) followed by saponification and then copper-catalyzed decarboxylation, which allow the preparation of **4** in a good overall yield (65% over two steps). Notably, if the decarboxylation step was performed in the presence of CuCN in dimethylacetamide, direct access to 4-cyano-5-methoxy-1*H*-indole (**5**) was obtained; otherwise, the same intermediate could also be prepared starting from **4** and using the same reaction.^31,32^

Pleasingly, the regioselective C3-alkylation of **4** and **5** proceeded uneventfully when *N*-acetyl ethanolamine was employed as the electrophile. It was conducted by taking advantage of the green (water being the only byproduct) borrowing hydrogen process using [{Cp*IrCl_2_}_2_] as the catalyst (2 mol %) and sodium *tert*-butoxide as the base in 42% yield for **6** and 31% yield for **7**.^33−36^ Suzuki cross-coupling between **7** and potassium vinyl trifluoroborate (vinylBF_3_K) was performed to install the vinyl group in good yield (88%). Oxidative cleavage of the C=C double bond to the corresponding 4CHO-MLT was conducted selectively over the undesired oxidation of the indole C_2_–C_3_ bond using the Lemieux–Johnson oxidation by employing a catalytic amount of osmium tetroxide and excess sodium periodate in the presence of a non-nucleophilic base, such as 2,6-lutidine. The use of inexpensive commercially available starting materials/reagents is the key feature of this optimized synthetic strategy.

**Figure 1:**
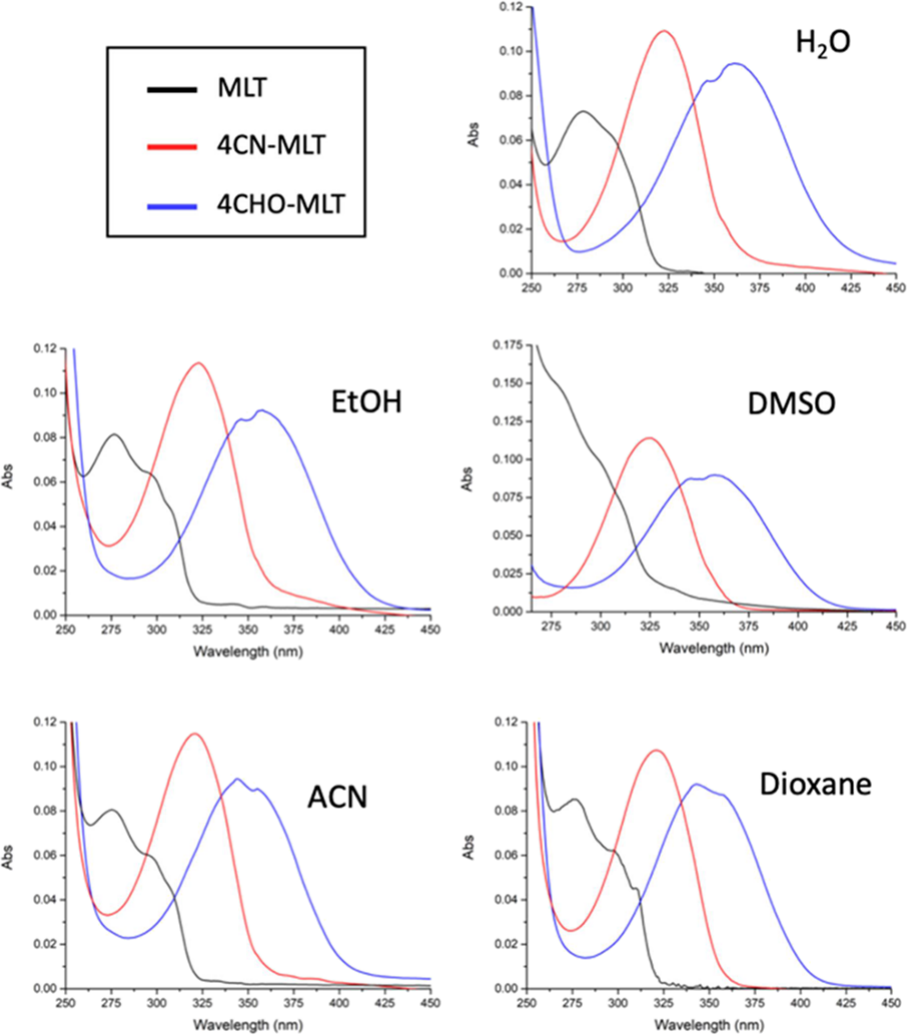
Absorption spectra of MLT, 4CN-MLT, and 4CHO-MLT, recorded at 10 μM in the indicated solvents.

With the synthetic compounds **6** and **9** in hand, we next investigated the optical properties of the synthesized derivatives in different solvents and compared them with those of MLT. A proof of concept of our investigation was gratifyingly obtained directly from the preparation of the target compounds. In fact, blue-light fluorescence could be observed with the naked eye upon illumination of 4CN-MLT with a UV lamp at a wavelength of 254 nm. The UV–Vis spectra of 4CN-MLT and 4CHO-MLT showed absorption bands at 324 and 362 nm, respectively, in aqueous solution (Figure 1).

Compared to that of MLT, the wavelengths at which 4CN-MLT and 4CHO-MLT had their strongest photon absorption (λ_max_) showed quite a strong red shift. This finding qualitatively confirmed the expected result that an electron-withdrawing group on an indole ring results in a red shift of λ_max_. Since the solvent affects the ground and excited state dipoles of a molecule, solvatochromic studies on 4CN-MLT and 4CHO-MLT were conducted in different aprotic and protic polar solvents, such as DMSO, ACN, EtOH, and 1,4-dioxane (Figure 1). No significant shifts in the absorption bands of **6** and **9** were observed in their UV–Vis spectra in these organic solvents. In particular, the λ_max_ of 4CHO-MLT showed a much greater red shift of up to 362 nm in water, whereas that of 4CN-MLT remained constant between 322 and 325 nm and was similar in the different solvents (Table 1).

**Table 1:** Affinity [*K*_i_ (nM)] Values of 4CN-MLT and 4CHO-MLT for the Human MLT Receptors MT1 and MT2

compound	MT_1_*K*_i_ (nM)	MT_2_*K*_i_ (nM)
4CN-MLT	2591 ± 168	398 ± 26
4CHO-MLT	1084 ± 79	424 ± 29
MLT	5.5 ± 0.4	6.3 ± 0.5

**Figure 2:**
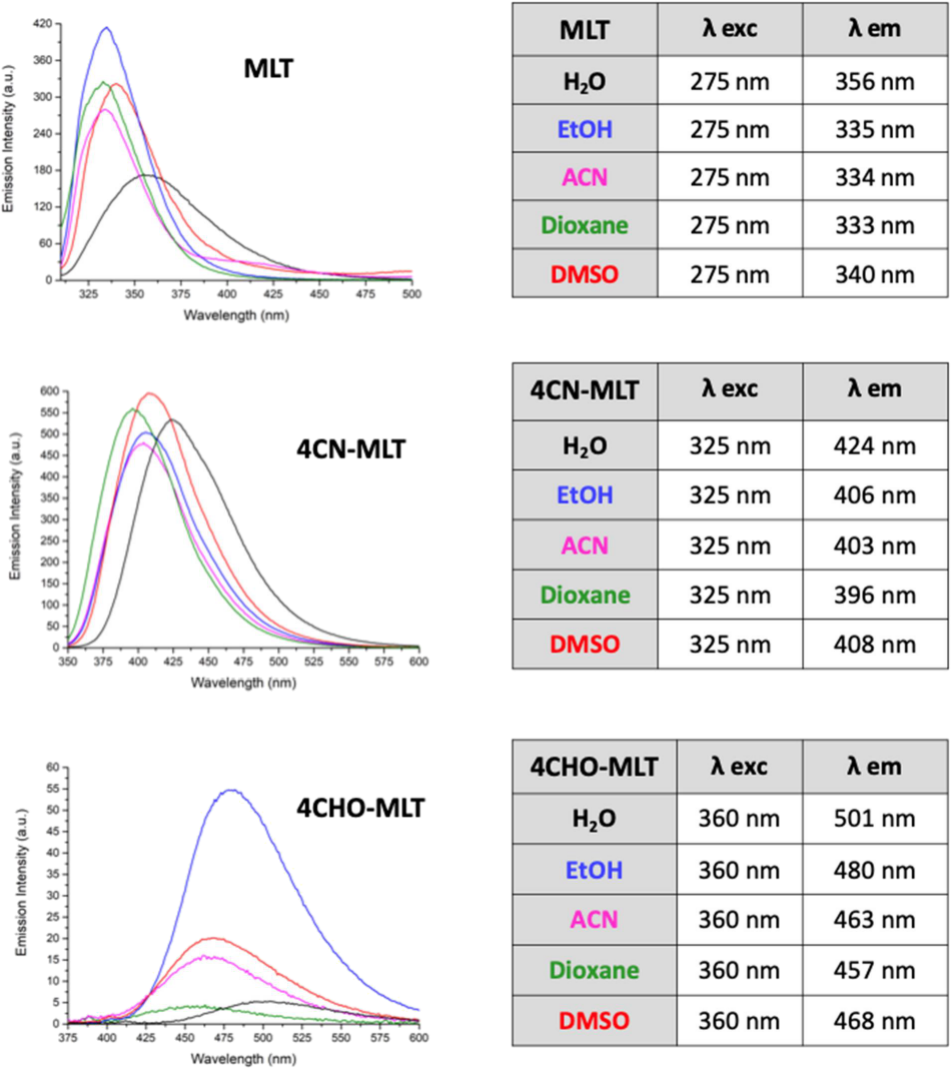
Emission spectra of MLT, 4CN-MLT, and 4CHO-MLT, recorded at 10 μM in all studied solvents.

When 4CN-MLT and 4CHO-MLT were excited at their λ_max_, their fluorescence properties were recorded. It was observed that the emission peaks of both 4CN-MLT and 4CHO-MLT were red shifted compared to that of MLT, as seen for their absorption peaks. Both compounds fluoresce in the visible spectral range, with 4CN-MLT whereas 4CHO-MLT fluoresces in the visible region near 500 nm showing a broad single emission band at around 410 nm (from 396 to 424 nm), whereas 4CHO-MLT. As a result, the absorption spectrum of 4CHO-MLT in ethanol extends beyond 400 nm, making the fluorescence excitable by blue light sources. In addition, the fluorescence spectrum of 4CHO-MLT, which peaks at approximately 480 nm in ethanol with a high intensity, indicates that this MLT derivative is a cyan fluorophore (Figure 2).

**Figure 3:**
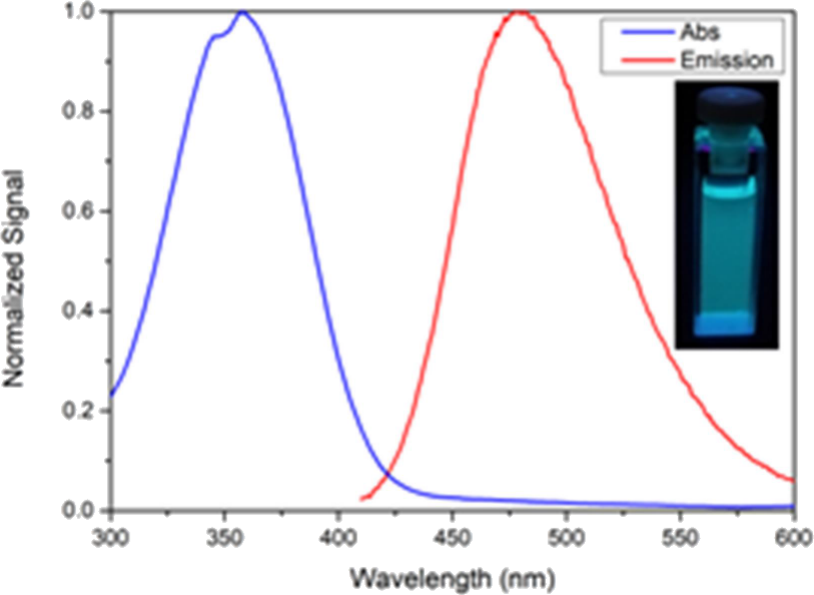
Normalized absorption (blue) and fluorescence (red) spectra of 4CHO-MLT (**9**) in ethanol. The excitation wavelength for the fluorescence measurement was 360 nm. As shown in the inset, 4CHO-MLT (**9**) is a cyan product.

For any molecule to be useful as a fluorescent probe for biological spectroscopy and microscopy experiments, other than selective excitation (beyond 360 nm), a strongly red-shifted fluorescence with a large Stokes shift (ca. 120–140 nm), and observable visible fluorescence, it must have a reasonably large fluorescence quantum yield, good photostability, and brightness. Therefore, we also characterized 4CHO-MLT in terms of these properties. As illustrated in the Supporting Information, 4CHO-MLT demonstrated a decent quantum yield (QY = 0.049), a suitable brightness (*B* = 1120 mM^–1^ cm^–1^), and a very good photostability (Figure 3).

Finally, it is worth noting that Ladner et al.^37^ have performed various photoreactions of the indole ring of tryptamine in chloroform to generate new tryptamine-based fluorophores. Interestingly, they found that one of the photoproducts exhibited a green color and a λ_max_ emission of 489 nm. In agreement, the small amount of product obtained corresponded to **9**.

Table 1 reports the affinity [*K*_i_ (nM)] values of 4CN-MLT and 4CHO-MLT for the human MLT receptors MT_1_ and MT_2_, which are stably expressed in rat fibroblast NIH3T3 cells, using 2-[^125^I]iodoMLT as the labeled ligand in competition binding assays. Compounds 4CN-MLT and 4CHO-MLT displayed modest affinity for both MLT receptors compared to MLT using a positive control.

As reported recently,^38,39^ the melatonin receptors contain a channel that provides the entrance route of ligands to the binding site from within the lipid bilayer. The atypical (related to metabolite serotonin in 5-HT receptors) entry mechanism could impose constraints on physicochemical properties (i.e., permanent dipole moment) and can be exploited in the future development of novel fluorescent melatonin-based structures to address the need for better binding. Specifically, we imagine that 4-dicyanovinylmelatonin would be a visible chromophore based on the fact that the 2,2-dicyanovinyl group is a stronger electronic withdrawing group than that of nitrile or formyl as well documented in the literature.^40^ In addition, 4CHO-MLT via its aldehydic formyl group could both complement and expand other modes of reactivity (e.g., oxime, hydrazone, and nitrone formation) to probe unusual and novel fluorescent behavior.

## 3 Conclusions

In summary, we have designed, synthesized, biologically evaluated, and surveyed the absorption and emission properties of two four-substituted MLT compounds, namely, 4CN-MLT and 4CHO-MLT, aiming to identify new biologically useful fluorescent melatoninergic ligands. This study provides a convenient means to identify compact MLT derivatives (which are expected to induce minimal perturbation to the native peptide structure, dynamics, or function), whose absorption spectra are red-shifted, and, importantly, whose fluorescence emits visible light. Extension of the π-conjugation of MLT at the C4 position by substitution with a formyl group induced a bathochromic shift to the visible region, and this derivative demonstrated a reasonably large fluorescence quantum yield and photostability. Although these novel compounds revealed only modest binding affinities to the MLT receptors MT1 and MT2, a new and reliable synthetic procedure for the preparation of novel and interesting C4-substituted indoles, which are widely studied by pharmaceutical chemists due to their unique biological activities, was reported.

## Supporting Information Available

Chemical synthetic methods and ^1^H and ^13^C NMR of target compounds and intermediates and spectroscopy (PDF)
