# Supplementary material for: Synthesis and Fluorescence Properties of 4-Cyano and 4-Formyl Melatonin as Putative Melatoninergic Ligands
Source: ACS Omega. 2023 Jun 8;8(24):22190–4. doi: 10.1021/acsomega.3c02518 (PMC10286092; doi:10.1021/acsomega.3c02518)
Supplement: Supplementary file 1 — ao3c02518_si_001.pdf [file ao3c02518_si_001.pdf]

Electronic Supplementary Information

Synthesis and Fluorescence Properties of 4-Cyano and 4-Formyl Melatonin as Putative Melatoninergic Ligands

Silvia Bartolucci,[§†] Michele Retini, [§†] Fabiola Fanini,[†] Daniele Paderni[‡] and Giovanni Piersanti*[†]

† *Department of Biomolecular Sciences*, University of Urbino Carlo Bo, Piazza Rinascimento 6, 61029 Urbino, PU, Italy
‡ *Departmentof Pure and Applied Sciences*, University of Urbino Carlo Bo, Via della Stazione 4, 61029 Urbino, PU, Italy
§ Contributed equally to this work.

* E-mail: giovanni.piersanti@uniurb.it
https://sites.google.com/uniurb.it/giovannipiersanti

Table of Contents

1. General Informations S3

2. Experimental Procedures S4

3. Absorption and Fluorescence Measurements S9

4. References S13

5. Copies of ^{1}H-NMR and ^{13}C-NMR spectra S14

1. General Informations

All reagents were purchased from best-known commercial suppliers and used without further purification, unless otherwise stated. All reactions were carried out under ambient condition, unless otherwise specified. Column chromatography purifications were performed in flash chromatography conditions using Merck 230-400 Mesh silica gel. Analytical thin layer chromatography (TLC) was carried out on Merck silica gel plates (Silica Gel 60 F_{254}), that were visualized by exposure to ultraviolet light and an aqueous solution of $KMnO_4$, p-anisaldehyde. ^{1}H-NMR and ^{13}C-NMR spectra were recorded on a Bruker Avance 400 spectrometer, using $CDCl_3$, $(CD_3)_2CO$, CD_3OD as solvent. NMR spectra were processed and analyzed using MestReNova software from Mestrelab Research. Chemical shifts (δ scale) are reported in parts per million (ppm) relative to the central peak of the solvent. Coupling constants (*J* values) are given in hertz (Hz). ESI-MS spectra were taken on a Waters Micromass ZQ instrument. Only molecular ions $[M+H]^+$ are given for the ESI-MS analysis. UV-Vis and fluorescence spectra. UV-Vis absorption spectra were recorded at 298.1 K on a Varian Cary-100 spectrophotometer equipped with a temperature control unit. Fluorescence emission spectra were recorded at 298.1 K on a Varian CaryEclipse spectrofluorimeter and the spectra are uncorrected. Emission quantum yields were calculated using 2,2′-biphenol in acetonitrile as standard ($\Phi = 0.29$). The HypSpec computer program was used to process the spectrophotometric data.

Binding affinities were determined using 2-[^{125}I]iodoMLT as the labelled ligand in competition experiments on cloned human MT_1 and MT_2 receptors expressed in NIH3T3 rat fibroblast cells. The characterization of NIH3T3-MT_1 and -MT_2 cells had been already described in detail.[1-2] Membranes were incubated for 90 min at 37 °C in binding buffer (Tris-HCl, 50 mM, pH 7.4). The final membrane concentration was 5–10 µg of protein per tube. The membrane protein level was determined in accordance with a previously reported method.[3] 2-[^{125}I]IodoMLT (100 pM) and different concentrations of MLT (10^{-10} – 10^{-6} M) or of the new compounds were incubated with the receptor preparation for 90 min at 37 °C. Nonspecific binding was assessed with 10 µM MLT; IC_{50} values were determined by nonlinear fitting strategies with the program PRISM (GraphPad Software Inc., San Diego, CA). The Ki values were calculated from the IC_{50} values in accordance with the Cheng– Prusoff equation.[4] The Ki values are the mean of at least three independent determinations performed in duplicate.

3. Experimental Procedures

Ethyl 4-bromo-5-methoxy-1*H*-indole-2-carboxylate 2:

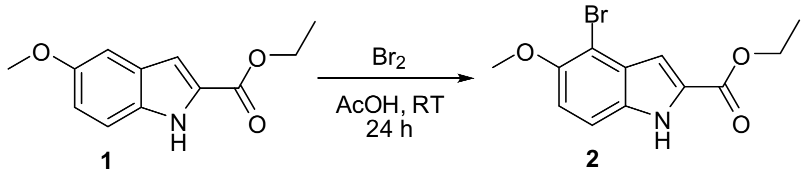

Scheme S1. Synthesis of compounds **2**

5-methoxy-l*H*-indole-2-carboxylate **1** (1 g, 4.57 mmol) was stirred with glacial acetic acid (22.9 mL) at 40 °C until only a small amount of indole remained undissolved. Bromine (234 µL, 4.57 mmol) was then added dropwise, maintaining 40 °C and a vigorous stirring (800-1000 rpm), and then allowed to stand (200 rpm) at ambient temperature for 24 h.

The resulting dark brown mixture was filtered, and the crystalline product was washed sequentially with acetic acid (2 x 4 mL) and cyclohexane (2 x 4 mL) and then dried at 45 °C in vacuo to give **2** (1.19 g, 3.98 mmol, 87%) as a white solid.

M.p.: 176 °C

MS (ESI): 298, 300 $[M+H]^+$

^{1}H-NMR ($CDCl_3$, 400 MHz): δ 1.44 (t, *J* = 7.1 Hz, 3H), 3.95 (s, 3H), 4.44 (q, *J* =7.1 Hz, 2H) 7.08 (d, *J* = 8.9 Hz, 1H), 7.24 (dd, *J* = 2.2 and 0.8 Hz, 1H), 7.34 (dd, *J* = 8.9 and 0.8 Hz, 1H), 9.10 (br s, 1H);

The chemical and physical data are in accord with those in the literature.[4]

4-bromo-5-methoxy-1*H*-indole-2-carboxylic acid 3:

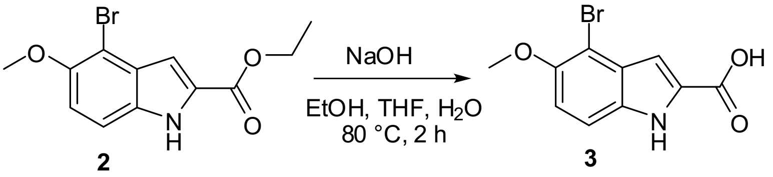

Scheme S2. Synthesis of compounds **3**

Ethyl 4-bromo-5-methoxy-1*H*-indole-2-carboxylate **2** (1.19 g, 3.98 mmol) was suspended in a mixture of THF (1.62 ml) and EtOH (6.5 ml). A 3 M aqueous solution of NaOH (3.24 ml) was added and the reaction was stirred for 2 h at 80 °C. The solution was cooled to 0 °C and stirred vigorously for 1 h as 2N aqueous solution of HCl was added (pH 2). The resulting thick yellow paste was filtered, and the crystalline product was washed with water (3 x 4 mL), and dried at 45 °C in vacuo to give the carboxylic acid **3** (1.05 g, 3.90 mmol, 98%).

M.p.: 261°C dec

MS (ESI): 268, 270 $[M-H]^-$

^{1}H-NMR (CD_3OD, 400 MHz): δ 3.89 (s, 3H), 7.06 (d, *J* = 0.8 Hz, 1H), 7.13 (d, *J* = 8.9 Hz, 1H), 7.40 (dd, *J* = 8.9 and 0.8 Hz, 1H);

The chemical and physical data are in accord with those in the literature.[4]

4-bromo-5-methoxy-1*H*-indole 4:

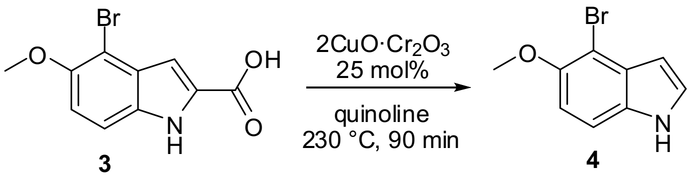

Scheme S3. Synthesis of compounds **4**

4-Bromo-5-methoxy-1*H*-indole-2-carboxylic acid **3** (1.05 g, 3.90 mmol), *freshly distilled* quinoline (14 ml) and copper chromite (barium-promoted) (303 mg, 0.975 mmol) were mixed together. Nitrogen was gently bubbled through the mixture for 5 minutes, then the mixture heated quickly to 220-230 °C under an atmosphere of nitrogen. After 90 minutes the mixture was cooled to ambient temperature diluted with ethyl acetate (120 ml) and 2 M aqueous solution of HCl (75 ml) and stirred for 10 minutes. The mixture was filtered through a pad of Celite, the filtrate dried with sodium sulphate and the solvent evaporated. The residue was purified by flash column chromatography on silica gel eluting with ciclohexane/ethyl acetate 8:2 to give 4-bromo-5-methoxy-1*H*-indole **4** (583 mg, 2.58 mmol, 66%).

MS (ESI): 226, 228 $[M+H]^+$

^{1}H-NMR ($CDCl_3$, 400 MHz): δ 3.94 (s, 3H), 6.57-6.59 (m, 1H), 6.94 (d, J = 8.7 Hz, 1H), 7.26-7.28 (m, 1H), 7.31 (dd, J = 8.7 and 0.9 Hz, 1H), 8.18 (br s, 1H)

The chemical and physical data are in accord with those in the literature.[5]

5-methoxy-1*H*-indole-4-carbonitrile 5:

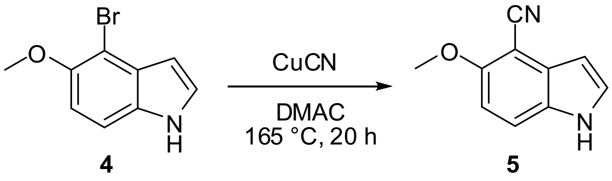

Scheme S4. Synthesis of compounds **5**

A 10-mL flask was charged with *freshly distilled N,N*-dimethylacetamide (4.3 mL), and the solvent was degassed (15 min) with a vigorous stream of nitrogen. 4-bromo-5-methoxy-1*H*-indole **4** (583 mg, 2.58 mmol) and copper cyanide (690 mg, 7.75 mmol) were added, and the solution was stirred at reflux (165 °C) for 20 h. The reaction mixture was cooled to room temperature, and poured into a mixture of water (5 mL) and ethyl acetate (5 mL) and filtered through a pad of Celite. The precipitate was washed with ethyl acetate (3 x 3 mL), and the combined organic phases were separated from the aqueous layer, washed with water (3 x 4 mL), and brine. The solution was dried and concentrated, and the yellow residue was recrystallized with ethyl acetate/hexane to give **5** (262 mg, 1.52 mmol, 59%) as light yellow solid (rods).

M.p.: 139-142°C

MS (ESI): 173 $[M+H]^+$

^{1}H-NMR ($CDCl_3$, 400 MHz): δ 3.97 (s, 3H), 6.66-6.67 (m, 1H), 6.89 (d, J = 8.9 Hz, 1H), 7.36-7.37 (m, 1H), 7.55 (dd, J = 8.9 and 0.8 Hz, 1H), 8.39 (br s, 1H);

The chemical and physical data are in accord with those in the literature.[4]

General procedure for borrowing hydrogen reaction:[7]

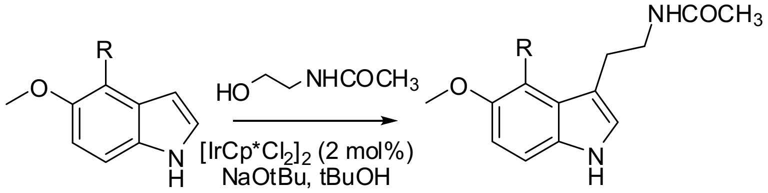

Scheme S5. Synthesis of compounds **6** and **7**

Under an air atmosphere, a 5 mL vial equipped with a stir bar, was sequentially charged with the appropriate indole **4** or **5** (1.5 equiv.), *N*-(2-hydroxyethyl)acetamide (1 equiv.), $[IrCp^*Cl_2]_2$ (2 mol%), NaO*t*Bu (0.5 equiv.) and *tert*-butanol (2.5 M). The reaction vessel was sealed and the vial was heated to 85 °C in a preheated oil bath for 24 h. The mixture was cooled to RT and filtered through a SiO_2 plug (eluting with ethyl acetate). The solvent was evaporated and the residue was purified by flash column chromatography on silica gel.

***N*-(2-(4-cyano-5-methoxy-1*H*-indol-3-yl)ethyl)acetamide 6:**

0.7 mmol scale reaction: 5-methoxy-1*H*-indole-4-carbonitrile **5** (180 mg, 1.05 mmol), *N*-(2-hydroxyethyl)acetamide (72 mg, 0.7 mmol), $[IrCp^*Cl_2]_2$ (11 mg, 0.014 mmol), NaO*t*Bu (34 mg, 0.35 mmol) and *tert*-butanol (1.75 mL) were subjected to general procedure for borrowing hydrogen reaction. Purification via column chromatography eluting with dichloromethane/acetone 8:2 afforded the title compound **6** as a white solid (75 mg, 0.29 mmol, 42%).

MS (ESI): 258 $[M+H]^+$

^{1}H-NMR (CD_3OD, 400 MHz): δ 1.92 (s, 3H), 3.11 (t, *J* = 7.2 Hz, 2H), 3.49 (t, *J* = 7.2 Hz, 2H), 3.95 (s, 3H), 6.97 (d, *J* = 9.0 Hz, 1H), 7.27 (s, 1H), 7.59 (d, *J* = 9.0 Hz, 1H)

^{13}C-NMR (CD_3OD, 400 MHz): δ 22.5, 25.7, 41.8, 57.4, 89.6, 107.3, 112.6, 118.4, 118.6, 128.3, 128.8, 133.5, 159.4, 173.3

HRMS (ESI-TOF) m/z calcd. for $C_{14}H_{15}N_3NaO_2$ $[M+Na]^+$: 280.1056; found 280.1067

***N*-(2-(4-bromo-5-methoxy-1*H*-indol-3-yl)ethyl)acetamide 7:**

1.3 mmol scale reaction: 4-bromo-5-methoxy-1*H*-indole **4** (440 mg, 1.95 mmol), *N*-(2-hydroxyethyl)acetamide (134 mg, 1.3 mmol), $[IrCp^*Cl_2]_2$ (21 mg, 0.026 mmol), NaO*t*Bu (62 mg, 0.65 mmol) and *tert*-butanol (3.25 mL) were subjected to general procedure for borrowing hydrogen reaction. Purification via column chromatography eluting with dichloromethane/methanol 97:3 afforded the title compound **7** as a beige foam (125 mg, 0.4 mmol, 31%).

MS (ESI): 311, 313 $[M+H]^+$

^{1}H-NMR ($CDCl_3$, 400 MHz): δ 1.96 (s, 3H), 3.25 (t, *J* = 6.9 Hz, 2H), 3.63 (dd, *J* = 12.9 and 6.9 Hz, 2H), 3.93 (s, 3H), 5.76 (br s, 1H), 6.93 (d, *J* = 8.8 Hz, 1H), 7.07 (d, *J* = 1.8 Hz, 1H), 7.27 (d, *J* = 8.8 Hz, 1H), 8.24 (br s, 1H)

The chemical and physical data are in accord with those in the literature.[8]

***N*-(2-(5-methoxy-4-vinyl-1*H*-indol-3-yl)ethyl)acetamide 8:**[9]

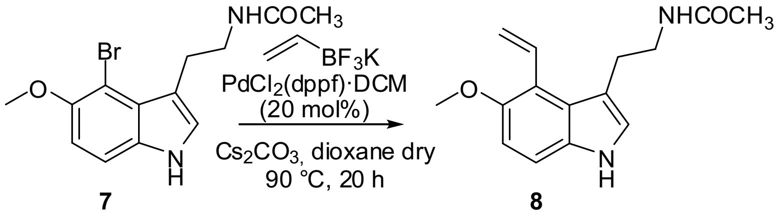

Scheme S6. Synthesis of compounds **8**

A 10 mL vial equipped with a stir bar was charged with *N*-(2-(4-bromo-5-methoxy-1*H*-indol-3-yl)ethyl)acetamide **7** (97 mg, 0.31 mmol), potassium vinyl trifluoroborate (83 mg, 0.62 mmol), Cs_2CO_3 (323 mg, 0.99 mmol), $PdCl_2(dppf)\cdot CH_2Cl_2$ complex (51 mg, 0.062 mmol) and subsequently 1,4-dioxane dry. The mixture was degassed (20 min) with a vigorous stream of Argon. The vial was transferred to an oil bath pre-heated to 90 °C and the reaction allowed to reflux under Argon for 20 h. The solution was allowed to cool to RT and diluted with ethyl acetate and water. The organic phase was separated and the acqueous phase was washed with ethyl acetate (3 x 50 mL). The combined organic phase was dried over Na_2SO_4, concentrated and the residue was purified by flash chromatography eluting with dichloromethane/methanol 97:3 afforded the title compound **8** (70 mg, 0.27 mmol, 88%) or use directly in the next reaction without purification.

MS (ESI): 259 $[M+H]^+$

^{1}H-NMR ($(CD_3)_2CO$, 400 MHz): δ 1.85 (s, 3H), 3.0 (t, *J* = 7.4 Hz, 2H), 3.42, (dt, *J* = 7.4 and 5.9 Hz, 2H), 3.80 (s, 3H), 5.48 (dd, *J* = 11.7 and 2.8 Hz, 1H), 5.77 (dd, *J* = 17.7 and 2.8 Hz, 1H), 6.91 (d, *J* = 8.8 Hz, 1H),7.07 (br s, 1H), 7.15 (d, *J* = 2.4 Hz, 1H), 7.21-7.28 (m, 2H), 9.94 (br s, 1H)

^{13}C-NMR ($(CD_3)_2CO$, 400 MHz): δ 23.0, 28.9, 41.1, 57.6, 110.2, 111.7, 113.8, 119.0, 119.9, 125.9, 126.6, 132.6, 134.3, 152.7, 169.7

HRMS (ESI-TOF) m/z calcd. for $C_{15}H_{18}N_2NaO_2$ $[M+Na]^+$: 281.1260; found 281.1269.

N-(2-(4-formyl-5-methoxy-1*H*-indol-3-yl)ethyl)acetamide 9:[9]

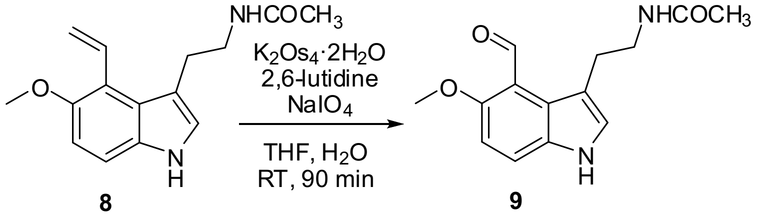

Scheme S7. Synthesis of compounds **9**

A vial equipped with a stir bar was charged with *N*-(2-(5-methoxy-4-vinyl-1*H*-indol-3-yl)ethyl)acetamide **8** (70 mg, 0.27 mmol) wich was subsequently dissolved using THF (3.8 mL) and H_2O (0.97 mL). To the stirring biphasic solution was added K_2OsO_4•$2H_2O$ (14 mg, 0.038 mmol), 2,6-lutidine (126 μL, 1.08 mmol) and $NaIO_4$ (179 mg, 0.84 mmol), respectively. The reaction was stirred at RT for 90 minutes before quenching with sat. aq. $NaHCO_3$ (15 mL) and then extracted with ethyl acetate (3 x 30 mL). The combined organic extracts were dried over Na_2SO_4 and concentrated under reduced pressure. Purification via column chromatography eluting with dichloromethane/methanol 97:3 afforded the title compound **9** as a amorphous light brown solid (18 mg, 0.07 mmol, 26%).

MS (ESI): 261 $[M+H]^+$

^{1}H-NMR ($(CD_3)_2CO$, 400 MHz): δ 1.83 (s, 3H), 3.13 (t, J = 7.1 Hz, 2H), 3.30-3.35 (m, 2H), 3.96 (s, 3H), 6.96 (br s, 1H), 7.01 (d, J = 8.9 Hz, 1H), 7.33 (d, J = 2.6 Hz, 1H), 7.68 (d, J = 8.9 Hz, 2H), 10.36 (br s, 1H), 10.69 (s, 1H)

^{13}C-NMR ($(CD_3)_2CO$, 400 MHz): δ 22.1, 28.2, 40.9, 56.8, 107.2, 114.5, 117.9, 119.0, 124.1, 128.5, 133.6, 159.5, 168.7, 190.6

HRMS (ESI-TOF) m/z calcd. for $C_{14}H_{16}N_2NaO_3$ $[M+Na]^+$: 283.1053; found 283.1055

4. Absorption and Fluorescence Measurements

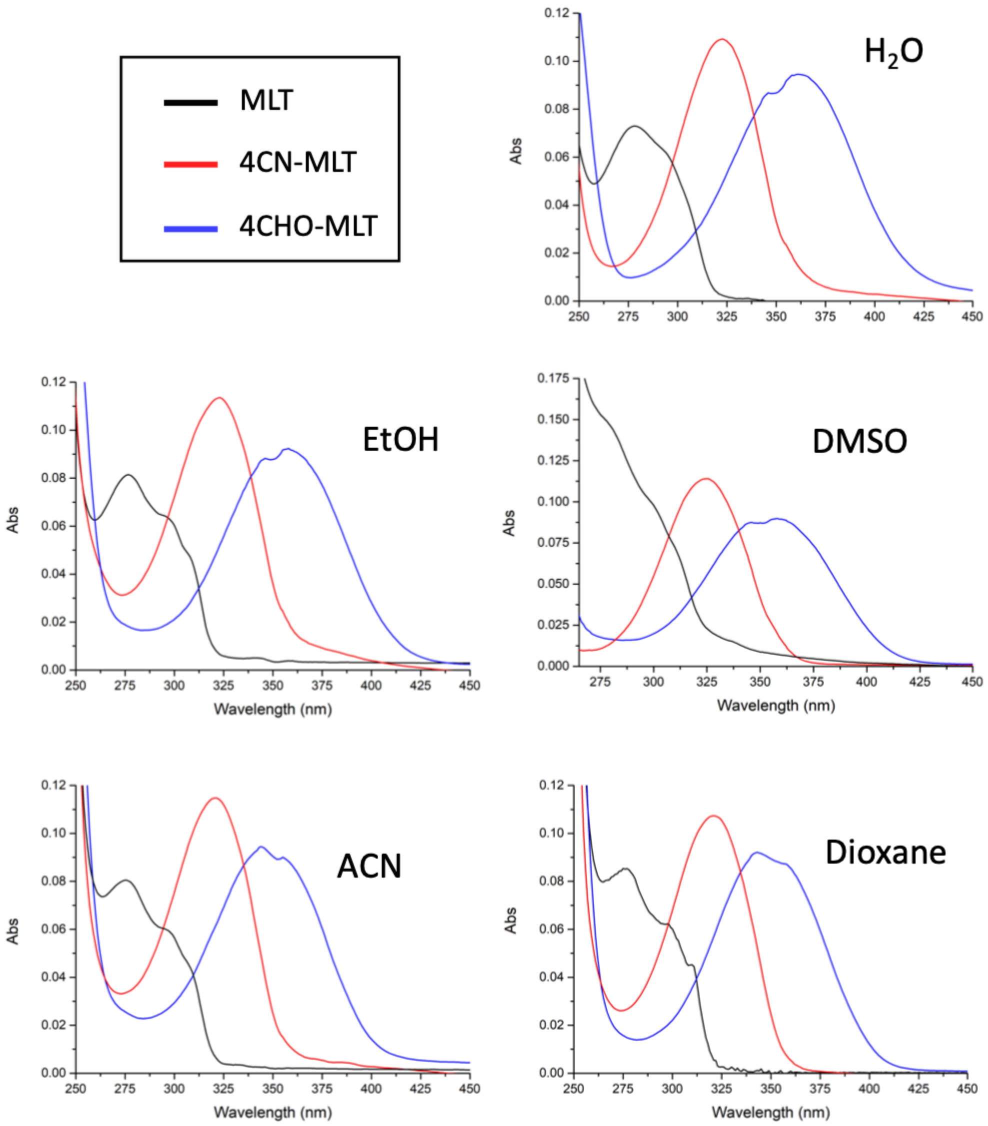

Figure S1. Absorption spectra of MLT, 4CN-MLT and 4CHO-MLT, recorded at 10 μM in the indicated solvents.

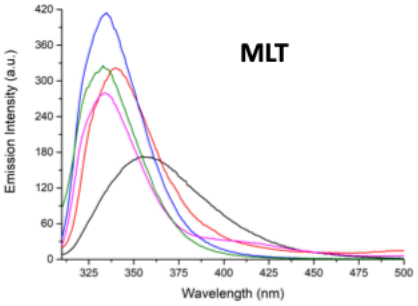

MLT	λ exc	λ em
H_2O	275 nm	356 nm
EtOH	275 nm	335 nm
ACN	275 nm	334 nm
Dioxane	275 nm	333 nm
DMSO	275 nm	340 nm

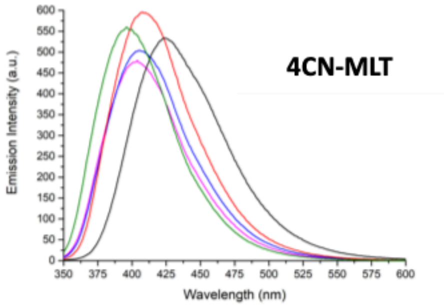

4CN-MLT	λ exc	λ em
H_2O	325 nm	424 nm
EtOH	325 nm	406 nm
ACN	325 nm	403 nm
Dioxane	325 nm	396 nm
DMSO	325 nm	408 nm

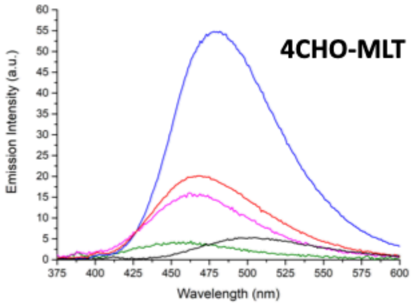

4CHO-MLT	λ exc	λ em
H_2O	360 nm	501 nm
EtOH	360 nm	480 nm
ACN	360 nm	463 nm
Dioxane	360 nm	457 nm
DMSO	360 nm	468 nm

Figure S2. Emission spectra of MLT, 4CN-MLT and 4CHO-MLT, recorded at 10 μM in all studied solvent.

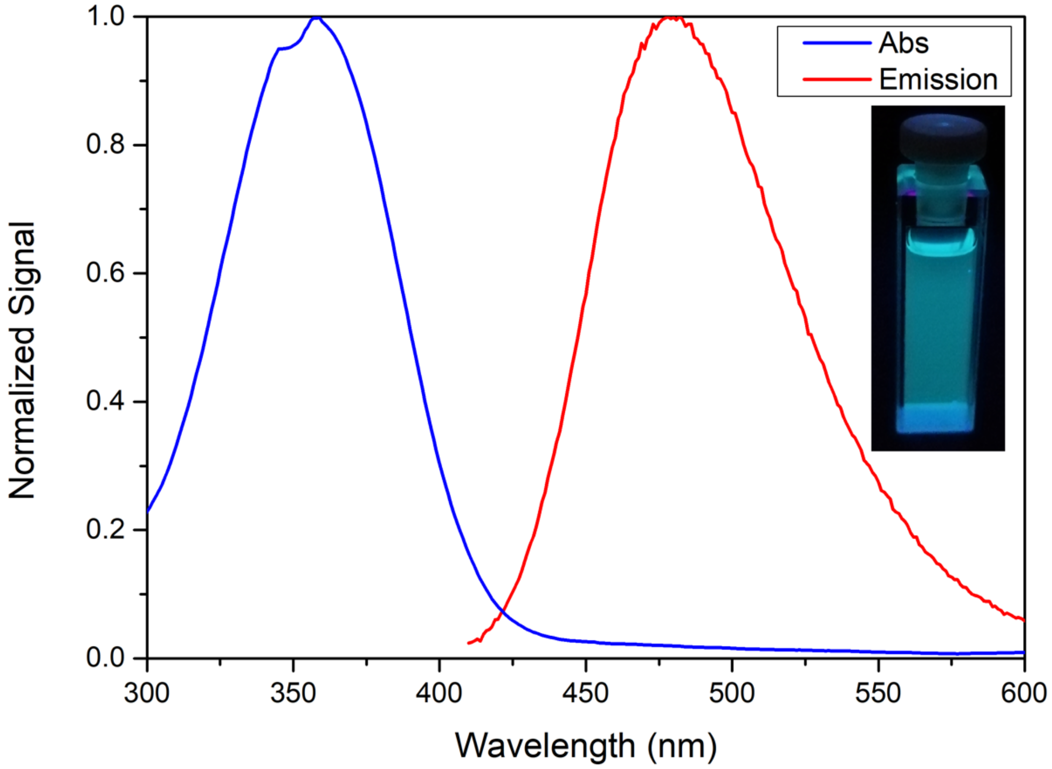

Figure S3. Normalized absorption (blue) and fluorescence (red) spectra of 4CHO-MLT (9) in ethanol. The excitation wavelength for the fluorescence measurement was 360 nm. As shown in the inset, 4CHO-MLT (9), is a cyan product.

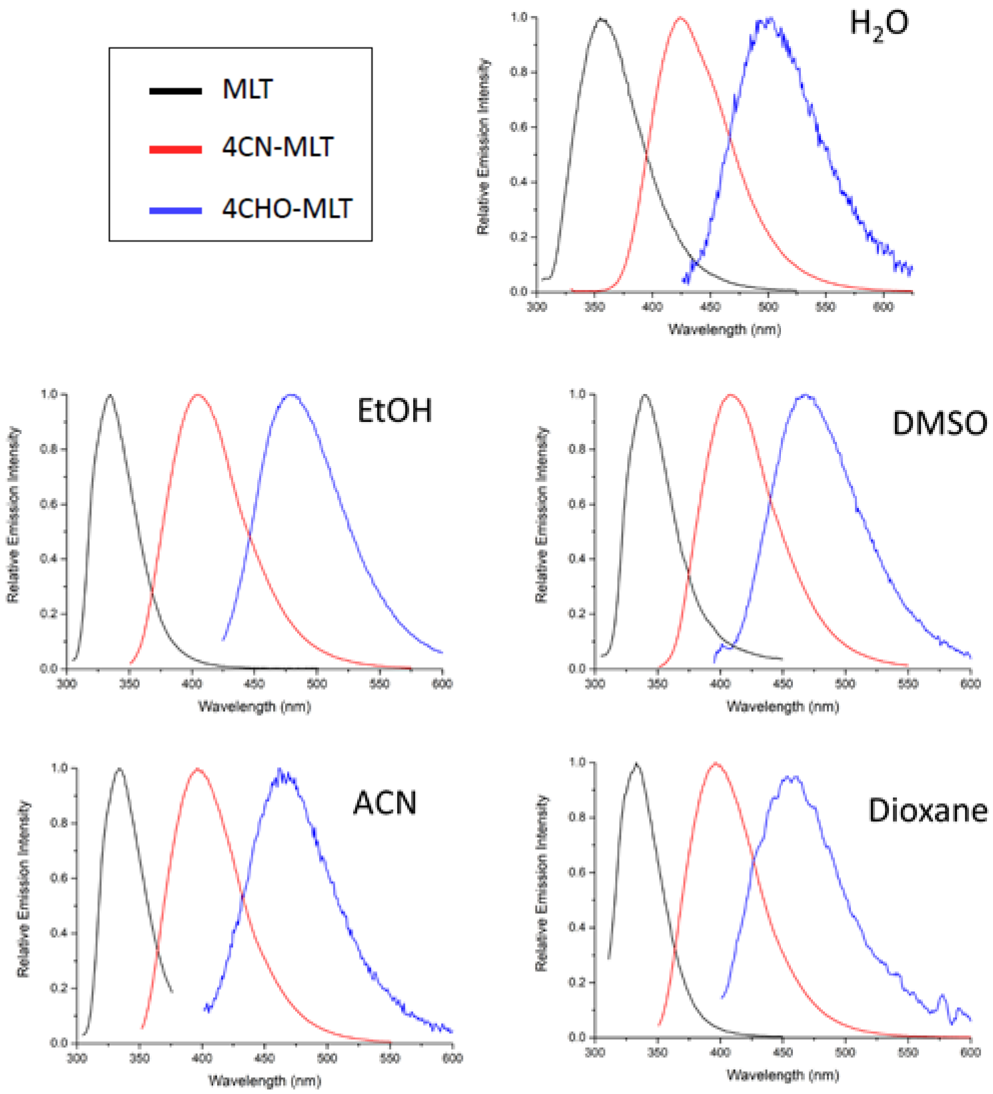

Figure S4. Fluorescence spectra of MLT, 4CN-MLT and 4CHO-MLT in the indicated solvent. For easy comparison, the fluorescence intensities in each panel were normalized.

Quantum yield (QY) and brightness determination:

QY was determined in ethanol at 20 °C using 2,2′-biphenol in acetonitrile as standard (Φ_R = 0.29).

$$QY = \Phi_R \cdot \frac{A_C}{A_R}\frac{n_C^2}{n_R^2} = 0{,}29 \cdot \frac{6233}{38008}\frac{1{,}3614^2}{1{,}3441^2} = 0{,}049$$

Brightness

$B = \varepsilon \cdot QY = 22900 \cdot 0{,}049 = 1120\ cm^{-1}M^{-1}$

5. References

(1) Nonno, R.; Lucini, V.; Pannacci, M.; Mazzucchelli, C. ; Angeloni, D.; Fraschini, F.; Stankov, B. M. Pharmacological characterization of the human melatonin Mel1a receptor following stable transfection into NIH3T3 cells, *Br. J. Pharmacol.* **1998**, *124*, 485–492.

(2) Spadoni, G.; Balsamini, C.; Bedini, A.; Diamantini, G.; Di Giacomo, B.; Tontini, A.; Tarzia, G.; Mor, M.; Plazzi, P.V.; Rivara, S.; Nonno, R.; Pannacci, M.; Lucini, V.; Fraschini, F.; Stankov, B.M. 2-[*N*-Acylamino(C1-C3)alkyl]indoles as MT_1 melatonin receptor partial agonists, antagonists, and putative inverse agonists, *J. Med. Chem.* **1998**, *41*, 3624–3634.

(3) Bradford, M.M. A rapid and sensitive method for the quantitation of microgram quantities of protein utilizing the principle of protein-dye binding, Anal. Biochem. **1976**, *72*, 248–254.

(4) Cheng, Y.; Prusoff, W.H. Relationship between the inhibition constant (Ki) and the concentration of inhibitor which causes 50% inhibition (IC_{50}) of an enzymatic reaction, *Biochem. Pharmacol.* **1973**, *22*, 3099–3108.

(5) Kruse, L. I.; Meyer, M. D. Ergoline synthons. 2. Synthesis of 1,5-dihydrobenz[cd]indol-4(3H)-ones and 1,3,4,5-tetrahydrobenz[cd]indol-4-amines. *J. Org. Chem.* **1984**, *49*, 4761−4768.

(6) Johansson, G.; Angbrant, J.; Ringom, R.; Hammer, K.; Ringberg, E.; Lindqvist, B.; Brandt, P.; Beierlein, K.; Nilsson, B. M. Indoles as 5-ht6 modulators. *Patent WO2008003703*, **2010**.

(7) Hall, C.J.J.; Goundry, W. R. F.; Donohoe, T. J. Hydrogen-Borrowing Alkylation of 1,2-Amino Alcohols in the Synthesis of Enantioenriched γ-Aminobutyric Acids. *Angew. Chem. Int. Ed.* **2021**, *60*, 6981-6985.

(8) Somei, M.; Fukui, Y.; Hasegawa, M.; Oshikiri, N.; Hayashi, T. Syntheses of Melatonin and Its Derivatives. *Heterocycles*, **2000**, *53*, 1725-1736.

(9) Micikas, R. J.; Ahmed, I. A.; Acharyya, A.; Smith III A. B.; Gai, F. Tuning the electronic transition energy of indole via substitution: application to identify tryptophan-based chromophores that absorb and emit visible light. *Phys. Chem. Chem. Phys.* **2021**, *23*, 6433−6437.

6. Copies of ^{1}H NMR and ^{13}C NMR spectra of synthesized compounds

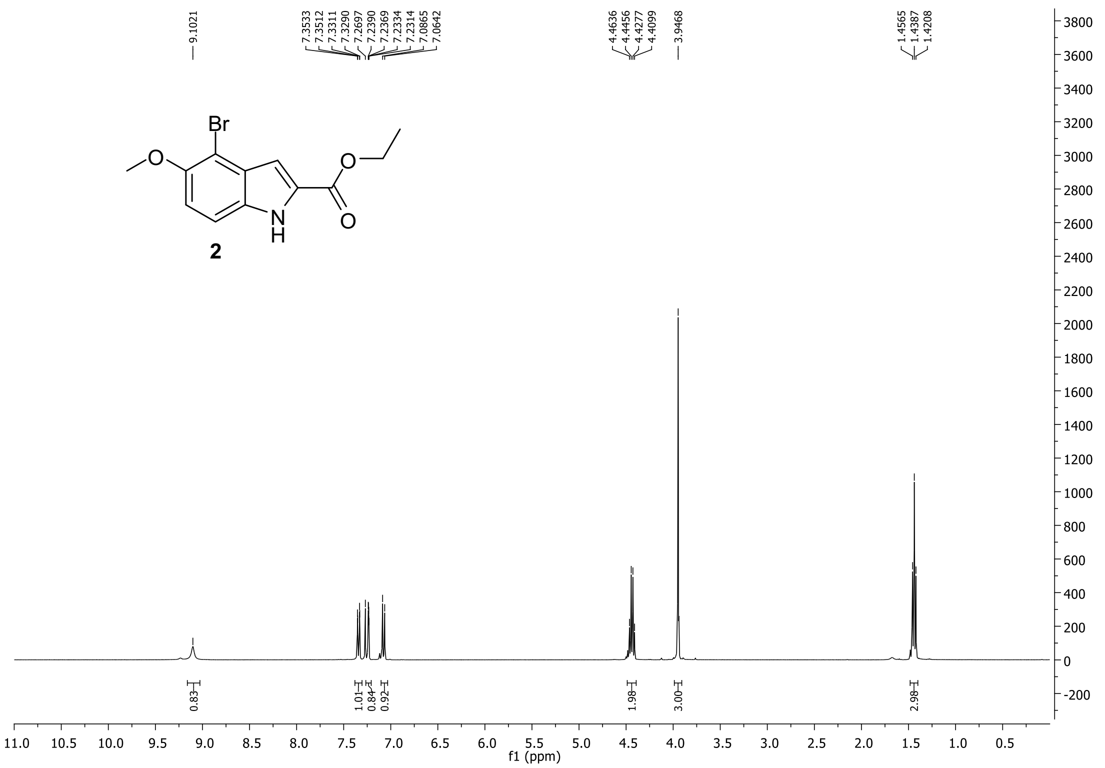

Figure S5. ^{1}H-NMR (400 MHz, $CDCl_3$) of ethyl 4-bromo-5-methoxy-1*H*-indole-2-carboxylate **2**

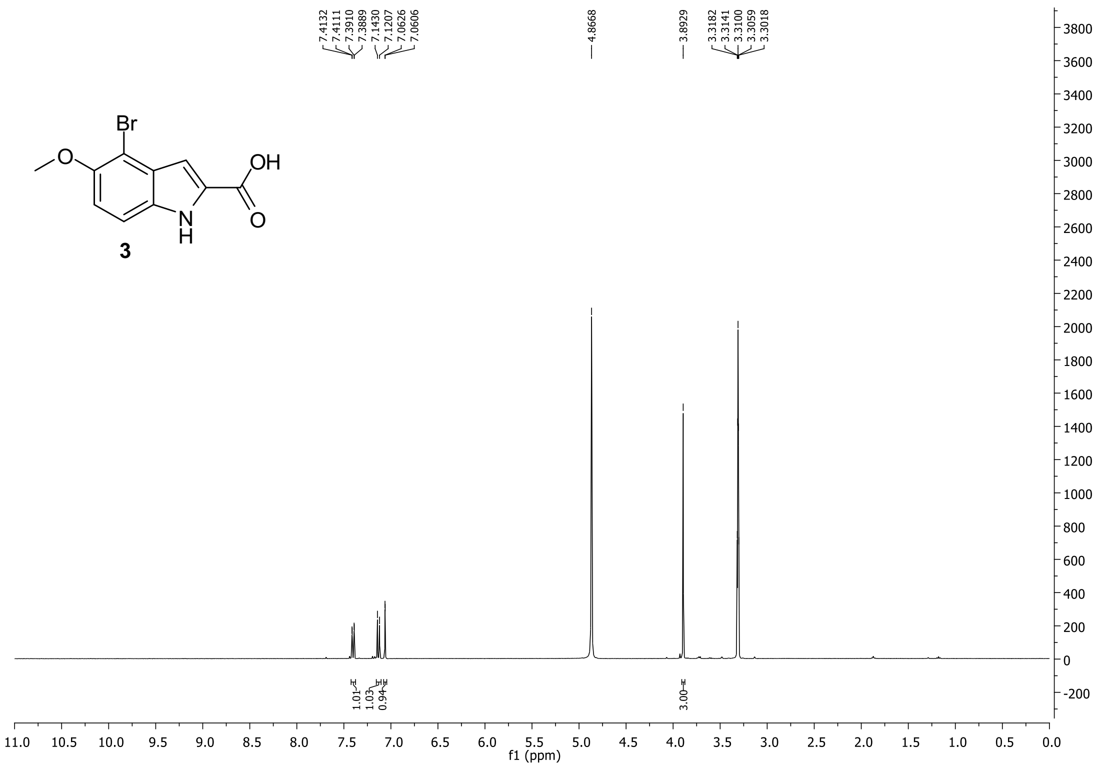

Figure S6. ^{1}H-NMR (400 MHz, CD_3OD) of 4-bromo-5-methoxy-1*H*-indole-2-carboxylic acid **3**

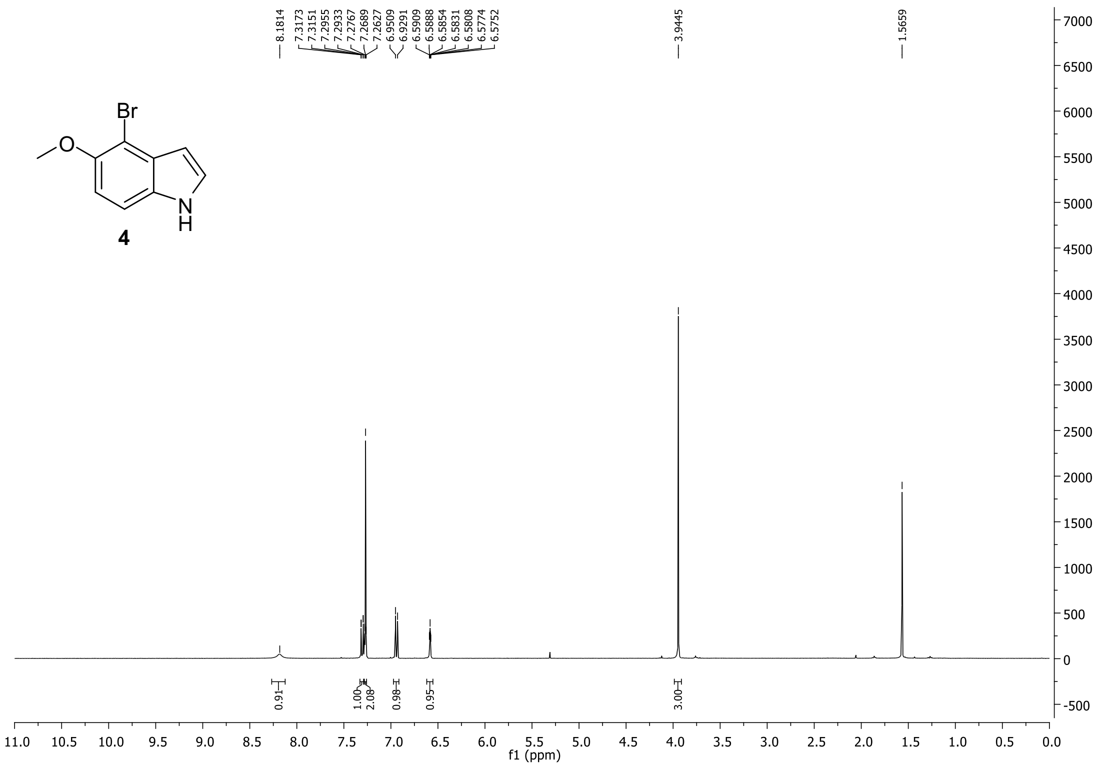

Figure S7. ^{1}H-NMR (400 MHz, $CDCl_3$) of 4-bromo-5-methoxy-1*H*-indole **4**

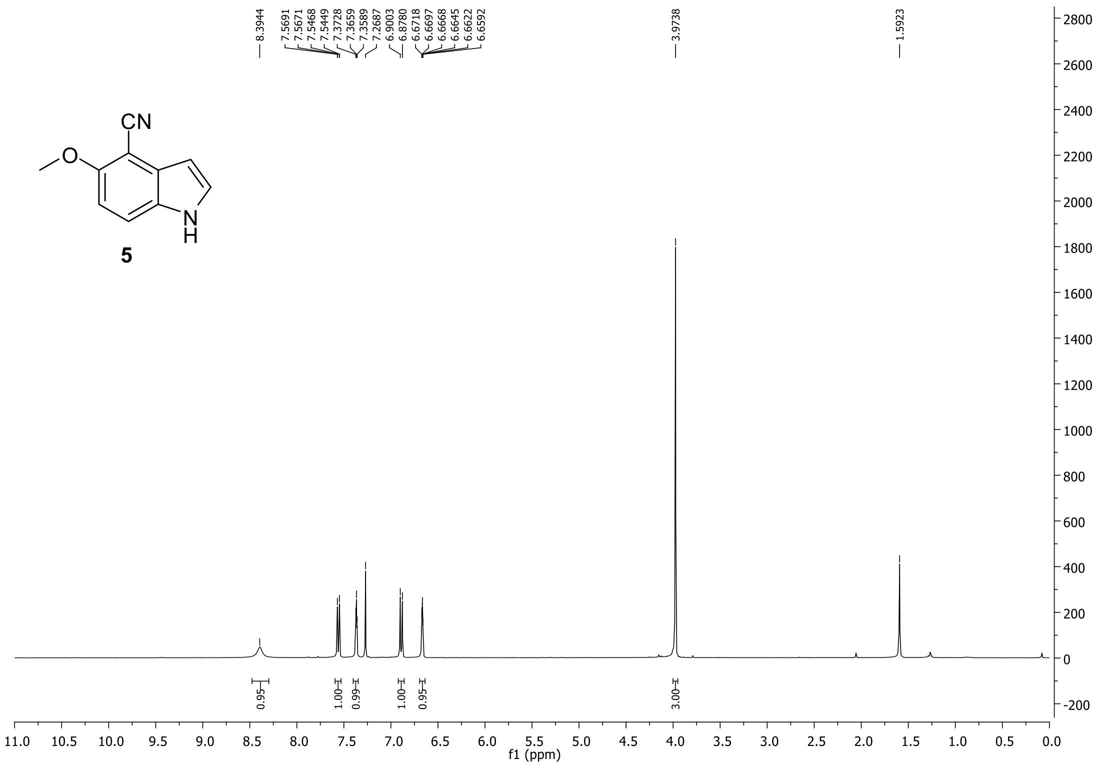

Figure S8. ^{1}H-NMR (400 MHz, $CDCl_3$) of 5-methoxy-1*H*-indole-4-carbonitrile **5**

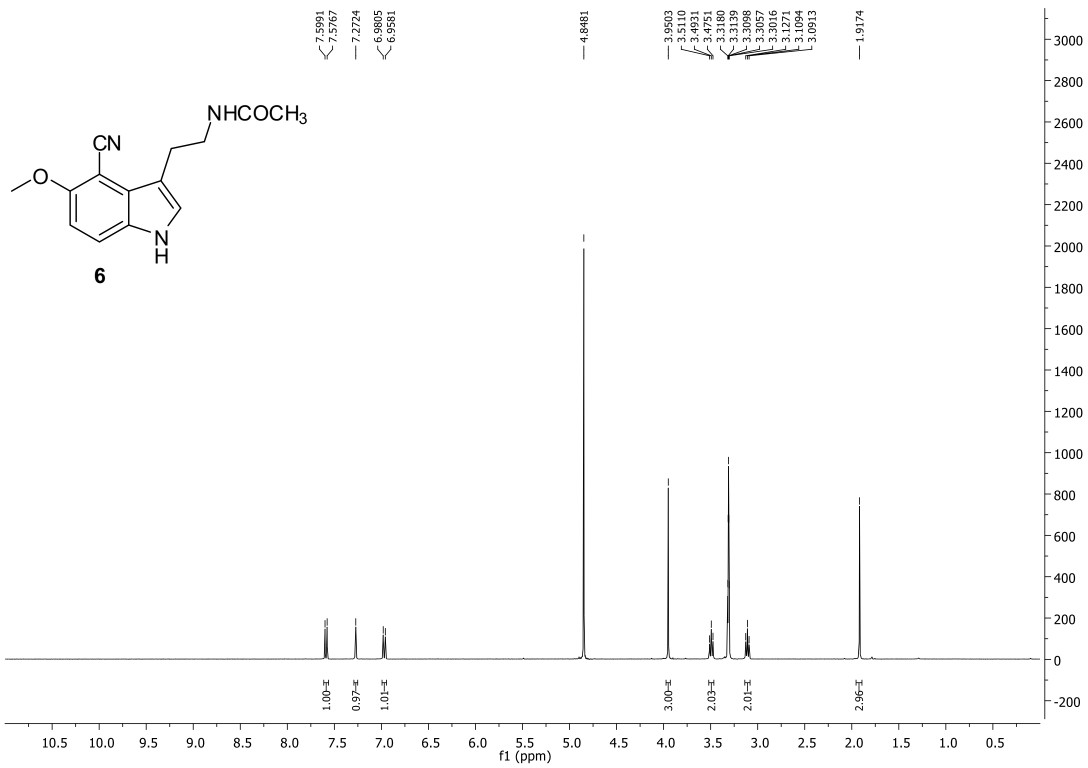

Figure S9. ^{1}H-NMR (400 MHz, CD_3OD) of *N*-(2-(4-cyano-5-methoxy-1*H*-indol-3-yl)ethyl)acetamide **6**

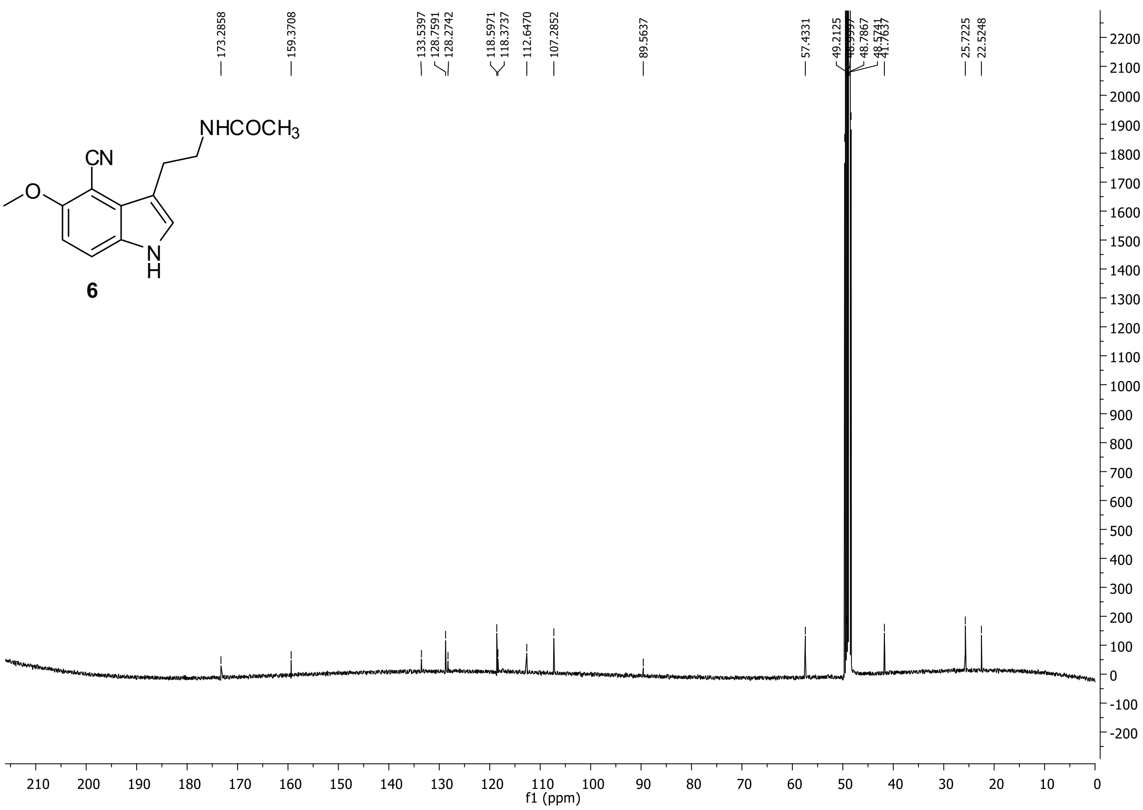

Figure S10. ^{13}C-NMR (200 MHz, CD_3OD) of *N*-(2-(4-cyano-5-methoxy-1*H*-indol-3-yl)ethyl)acetamide **6**

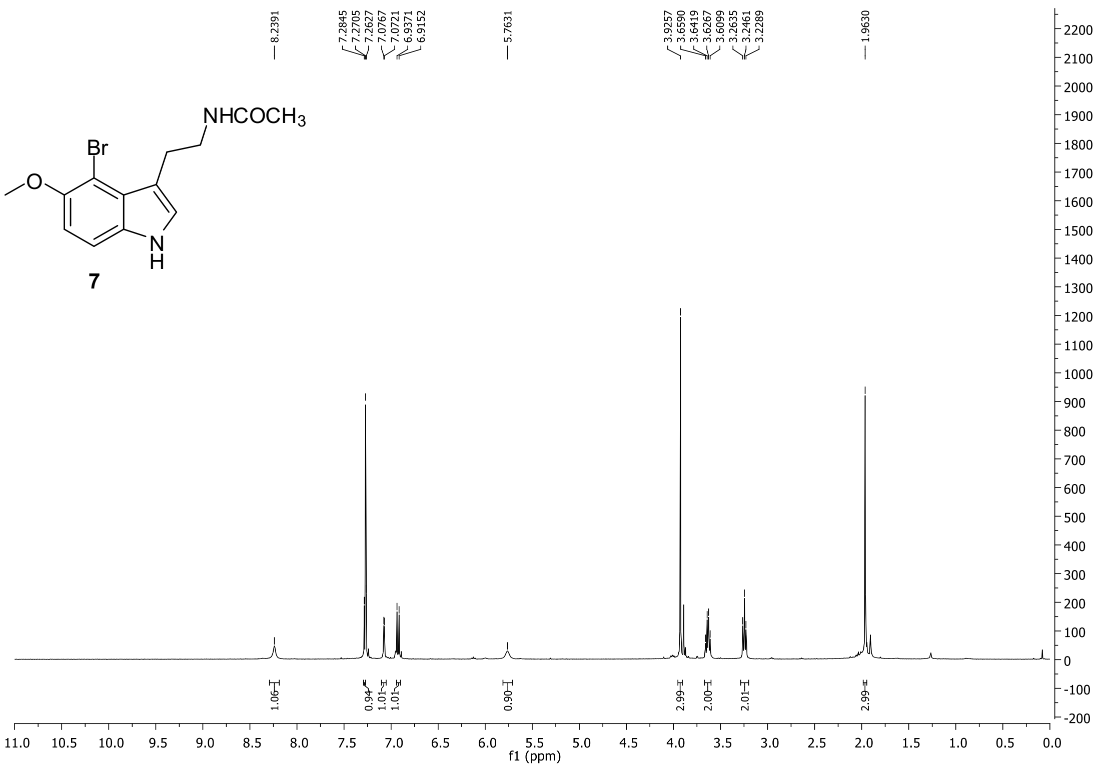

Figure S11. ^{1}H -NMR (400 MHz, $CDCl_3$) of *N*-(2-(4-bromo-5-methoxy-1*H*-indol-3-yl)ethyl)acetamide **7**

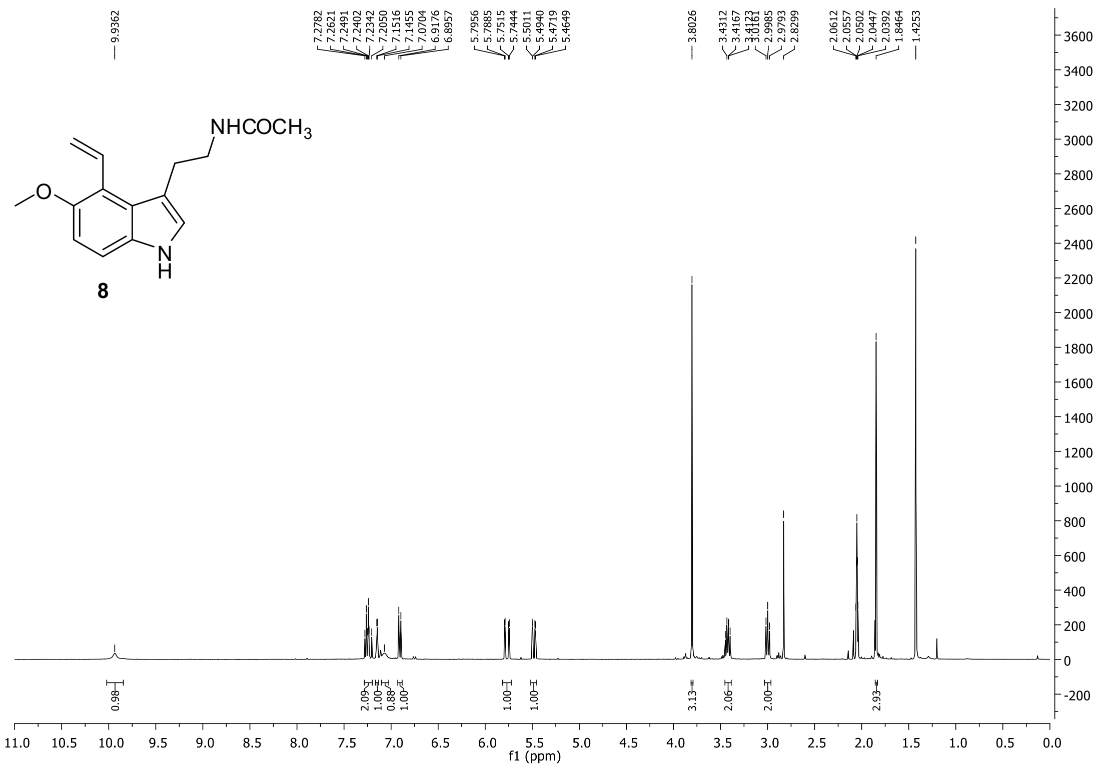

Figure S12. ^{1}H -NMR (400 MHz, $(CD_3)_2CO$) of *N*-(2-(5-methoxy-4-vinyl-1*H*-indol-3-yl)ethyl)acetamide **8**

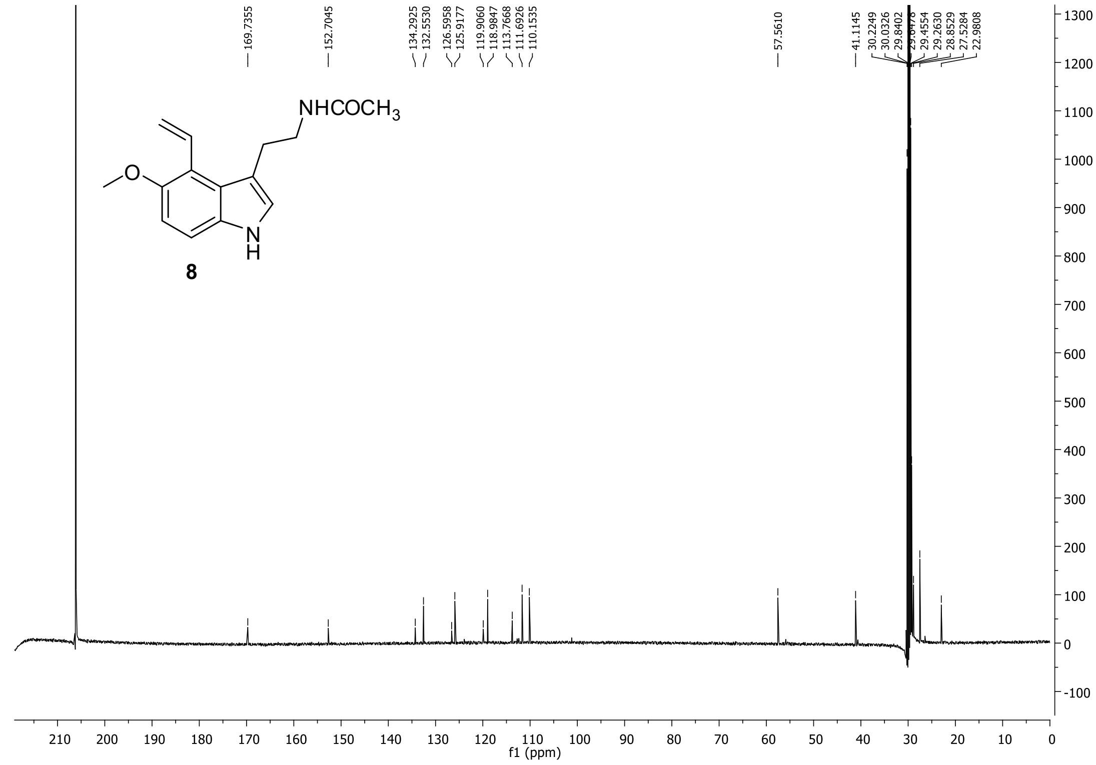

Figure S13. ^{13}C -NMR (200 MHz, $(CD_3)_2CO$) of *N*-(2-(5-methoxy-4-vinyl-1*H*-indol-3-yl)ethyl)acetamide **8**

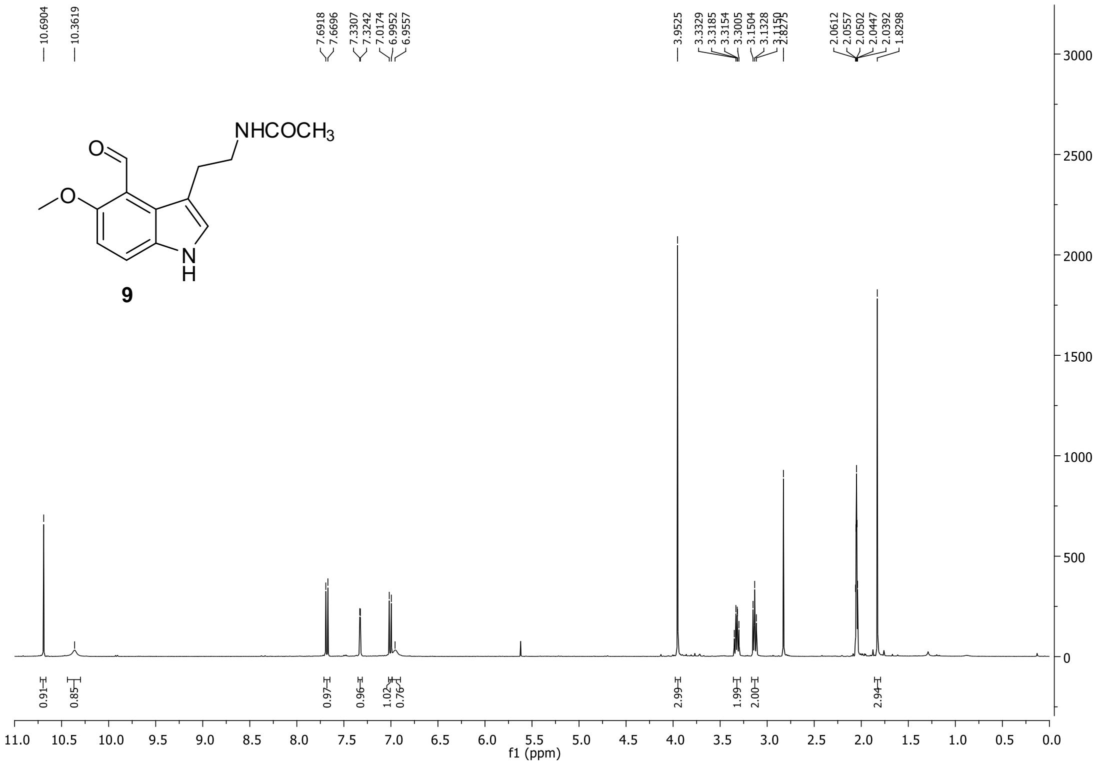

Figure S14. ^{1}H -NMR (400 MHz, $(CD_3)_2CO$) of *N*-(2-(4-formyl-5-methoxy-1*H*-indol-3-yl)ethyl)acetamide **9**

^{13}C-NMR of *N*-(2-(4-formyl-5-methoxy-1*H*-indol-3-yl)ethyl)acetamide 9:

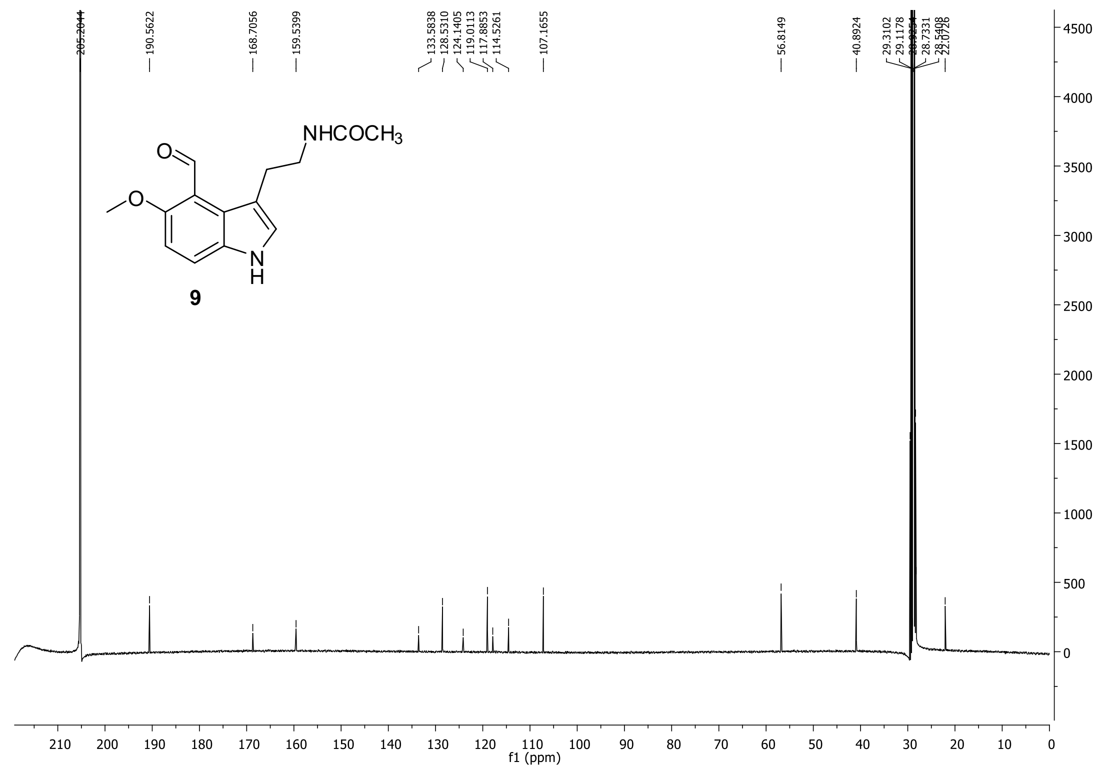

Figure S15. ^{13}C -NMR (200 MHz, $(CD_3)_2CO$) of *N*-(2-(4-formyl-5-methoxy-1*H*-indol-3-yl)ethyl)acetamide **9**
